# The Post-Translational Modifications of Human Salivary Peptides and Proteins Evidenced by Top-Down Platforms

**DOI:** 10.3390/ijms241612776

**Published:** 2023-08-14

**Authors:** Irene Messana, Barbara Manconi, Tiziana Cabras, Mozhgan Boroumand, Maria Teresa Sanna, Federica Iavarone, Alessandra Olianas, Claudia Desiderio, Diana Valeria Rossetti, Federica Vincenzoni, Cristina Contini, Giulia Guadalupi, Antonella Fiorita, Gavino Faa, Massimo Castagnola

**Affiliations:** 1 Istituto di Scienze e Tecnologie Chimiche “Giulio Natta”, Consiglio Nazionale delle Ricerche, 00168 Rome, Italy; imessana53@gmail.com (I.M.); claudia.desiderio@cnr.it (C.D.); dianaross79@hotmail.com (D.V.R.); 2 Department of Life and Environmental Sciences, University of Cagliari, 09124 Cagliari, Italy; bmanconi@unica.it (B.M.); sanna@unica.it (M.T.S.); olianas@unica.it (A.O.); cristina.contini93@unica.it (C.C.); giulia.guadalupi@unica.it (G.G.); 3 National Institut on Aging, NIH, Baltimore, MD 21224, USA; mozhgan.boroumand@nih.gov; 4 Dipartimento di Scienze Biotecnologiche di Base, Cliniche Intensivologiche e Perioperatorie, Università Cattolica del Sacro Cuore, 00168 Rome, Italy; federica.iavarone@unicatt.it (F.I.); federica.vincenzoni@unicatt.it (F.V.); 5 Fondazione Policlinico Universitario A. Gemelli Fondazione IRCCS, 00168 Rome, Italy; antonella.fiorita@policlinicogemelli.it; 6 Dipartimento di Scienze dell’Invecchiamento, Neurologiche, Ortopediche e della Testa e del Collo, Università Cattolica del Sacro Cuore, 00168 Rome, Italy; 7 Unit of Pathology, Department of Medical Sciences and Public Health, University of Cagliari, 09124 Cagliari, Italy; gavinofaa@gmail.com; 8 Department of Biology, College of Science and Technology, Temple University, Philadelphia, PA 19122, USA; 9 Proteomics Laboratory, European Center for Brain Research, (IRCCS) Santa Lucia Foundation, 00168 Rome, Italy; maxcastagnola@outlook.it

**Keywords:** salivary proteins, top-down proteomics, post-translational modifications

## Abstract

In this review, we extensively describe the main post-translational modifications that give rise to the multiple proteoforms characterized to date in the human salivary proteome and their potential role. Most of the data reported were obtained by our group in over twenty-five years of research carried out on human saliva mainly by applying a top-down strategy. In the beginning, we describe the products generated by proteolytic cleavages, which can occur before and after secretion. In this section, the most relevant families of salivary proteins are also described. Next, we report the current information concerning the human salivary phospho-proteome and the limited news available on sulfo-proteomes. Three sections are dedicated to the description of glycation and enzymatic glycosylation. Citrullination and N- and C-terminal post-translational modifications (PTMs) and miscellaneous other modifications are described in the last two sections. Results highlighting the variation in the level of some proteoforms in local or systemic pathologies are also reviewed throughout the sections of the manuscript to underline the impact and relevance of this information for the development of new diagnostic biomarkers useful in clinical practice.

## 1. Introduction

Human whole saliva is a hypotonic fluid lining the oral cavity and is composed of water (99%) and a complex mixture of organic and inorganic compounds resulting from salivary gland secretion, oral flora, the oropharynx, the upper airway, gastrointestinal reflux, gingival crevicular fluid, food deposits, and mucosal surface secretion containing blood-derived components [1]. Saliva is essential for the accomplishment of multiple physiological functions, encompassing lubrication, buffering, the maintenance of tooth integrity, chewing, the initial digestion of some foods, swallowing, tissue hydration and lubrication, speech, and wound healing, and it exhibits antibacterial and antifungal activity [2]. The dramatic sequelae observed in patients suffering from Sjögren syndrome clearly demonstrate the relevance of saliva and its components, particularly salivary proteins, in the protection of the mouth. Recent proteomic inventories report more than 3000 proteoforms in human saliva [3]. Before, during, and after secretion, most salivary proteins undergo numerous post-translational modifications (PTMs), of which the roles have not yet been clearly elucidated. Over the last twenty-five years, our group has been able to characterize many PTMs of the salivary proteome, mainly by applying top-down proteomic platforms to saliva from healthy subjects of various ages and from patients affected by various pathologies. In this review, we report the PTMs most frequently observed, suggesting, when possible, some hypotheses on the possible role played in the protection of the mouth. We have conducted exhaustive research on these topics in the literature. Nonetheless, we apologize in advance for any possible omission.

## 2. Enzymatic Cleavages (Cryptides)

The most-common PTM detectable in human saliva is the proteolytic cleavage of proteins. Indeed, most salivary proteins are submitted to the action of endogenous and exogenous proteinases (the last mainly from oral flora), which leads to the formation of a myriad of fragments. Many of these fragments could be considered important members of the cryptide family, defined as bioactive peptides encrypted inside a bigger functional polypeptide and released by a proteolytic event, with distinct or related function, but not superimposable, to that of the parental polypeptides [4]. The action of proteases can occur before and after secretion, and in the following paragraphs, this topic will be reviewed by describing pre-secretory and post-secretory cleavages separately.

### 2.1. Pre-Secretory Cleavages

**Figure 1:**
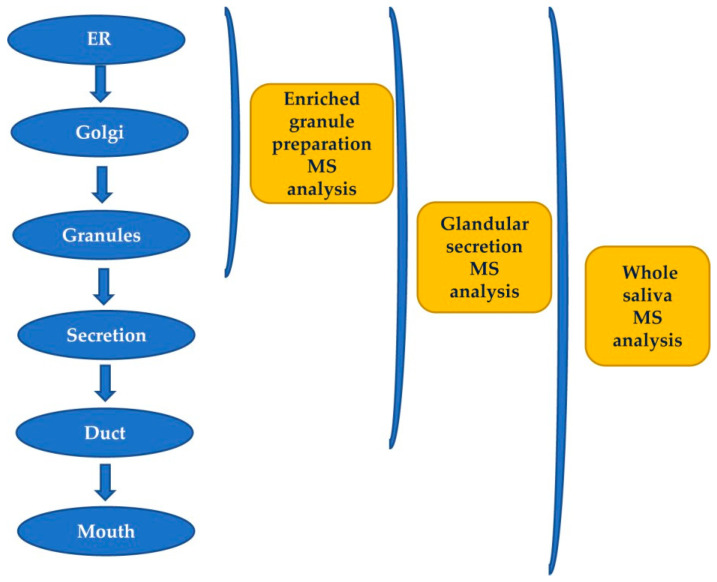
Workflow applied for studying the origin of post-translational modifications (PTMs) of salivary peptides and proteins secreted by the major salivary glands by a top-down proteomic approach.

The secretory pathway for many proteins includes transit in the Golgi apparatus and storage in secretory granules preceding their release from the cell into the duct system and secretion into the mouth [5]. Most of the pre-secretory cleavages of proteins occur during the transport towards the granules of the trans-Golgi-network [6], which represents the major secretory-pathway sorting station. Glandular secretions, protein extracts from secretory granules isolated from the major salivary glands, and whole saliva were investigated by top-down proteomics to characterize and distinguish between events occurring prior to the storage in the secretory granules, those taking place between granule release and secretion into the mouth during the passage through the secretory duct, and those occurring into the mouth [6]. The workflow applied in that study is represented in Figure 1. Proteases involved in the most relevant cleavages belong to the convertase and exopeptidase families [6,7,8,9].

#### 2.1.1. Proline-Rich Proteins (PRPs)

Human PRPs constitute a very polymorphic family of proteins, missing amino acids in their sequence aromatic and characterized by a high content of proline (as well as glutamine and glycine). All PRPs are encoded by chromosome 12p13.2 and are divided into three classes based on their ionic properties, i.e., acidic proline-rich-proteins (aPRPs), encoded by PRH1 and PRH2 loci; basic proline-rich proteins (bPRPs), encoded by the PRB1, PRB2, and PRB4 loci located (considering the direction of translation) immediately before the aPRPs loci; and the glycosylated (basic) proline-rich proteins (gPRPs), encoded by the PRB3 locus, which is the fourth locus of the bPRPs cluster. It is relevant to underline that except for the bPRPs named P-D, the other three proteoforms of the PRB-4 locus, i.e., the IB-8a (Con 1+) of PRB-2 and all the products of the PRB-3 locus, are submitted to glycosylation, which will be described in more detail, if available, in the section devoted to this PTM.

##### Acidic Proline-Rich-Proteins (aPRPs)

**Figure 2:**
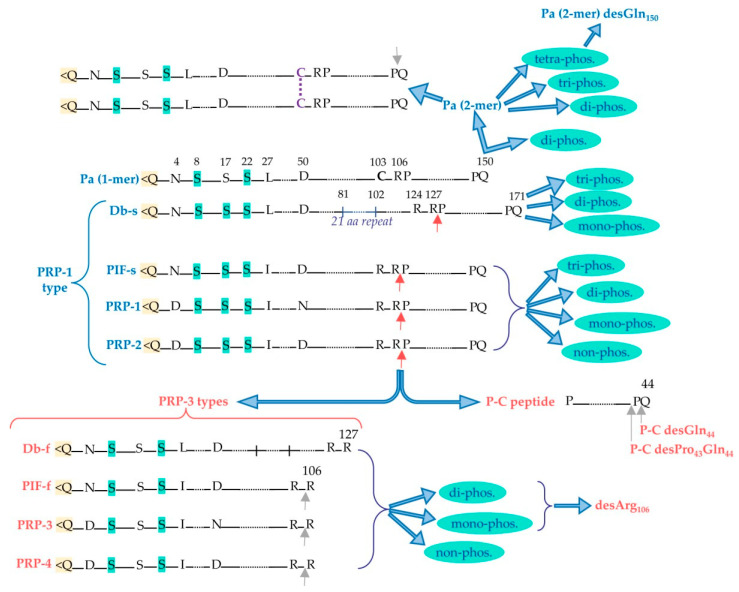
Principal acidic proline-rich protein (aPRP) proteoforms: in blue are the entire proteoforms and in red are the truncated ones. Red arrows indicate the cleavage site recognized by convertase activity, from which PRP-3-type truncated forms and the P-C peptide are generated from the entire PRP-1-type forms. Grey arrows indicate the possible loss of C-terminal residue by carboxypeptidase activity. Pyroglutamination N-terminal is evidenced in yellow, while the Ser residues that can undergo phosphorylation are evidenced in light green. The different phosphorylated proteoforms obtainable from the entire and truncated aPRPs are shown, as well as the disulfide dimeric form of Pa.

aPRPs exist as five principal isoforms called PRP-1, PRP-2 (coded by *PRH-2* locus) and Pif-s (parotid isoelectric-focusing isoform-slow), Pa (parotid acidic isoform), and Db-s (double band isoform-slow) (coded by *PRH-1* locus). The isoforms PRP-1, PRP-2, Pif-s, and Pa are all 150 residues long. Taking as reference PRP-2, the Pif-s and Pa isoforms have an asparagine (Asn_4_) instead of Asp_4_, and PRP-1 has Asn_50_ instead of the Asp_50_ present in the other isoforms (Figure 2). The Db-s isoform is 171 a.a. residues long due to the insertion of 21 residues repeated after position 81 and, as Pa, it has a Leu_27_ instead of Ileu_27_. The PRP-1, PRP-2, and PIF-s isoforms are partially cleaved at the Arg_106_ by a convertase recognizing the …R_103_XXR_106_… consensus sequence, generating the PRP-3, PRP-4, and Pif-f (f stands for fast electrophoretic isoform) truncated isoforms and a terminal fragment of 44 amino-acid residues called the P-C (or IB-8b) peptide. The convertase consensus sequence of Db-s is shifted by the insertion, and it is cleaved at Arg_127_, generating the Db-f isoform and always the P-C peptide. The consensus sequence of the convertase responsible for the cleavage is preserved because the Pa isoform, having a Cys_103_ residue instead of Arg_103_, is not cleaved. Indeed, a truncated Pa isoform was never detected. Rather, it generates in the mouth a Pa-2-mer isoform due to the formation of a disulfide bridge between two Cys_103_ residues (Figure 2), which is the main Pa component detectable in whole saliva [9]. The cleavage of PRP-1, PRP-2, Pif-s, and Db-s is not complete, and in whole saliva, the entire and truncated isoforms are both detectable, approximatively in a proportion of 70/30%, respectively. All the entire isoforms are the PRP-1 type, while all the truncated are the PRP-3 type (obviously non-including the Pa proteoform). All these characteristics of aPRPs are summarized in Figure 2.

##### Basic-Proline-Rich-Proteins (bPRPs)

bPRPs are encoded by the polymorphic *PRB1*, *PRB2,* and *PRB4* loci, existing as three or four possible alleles: (S(mall), M(edium), and L(arge), or for the eventual fourth allele, V(ery)L(arge)). Unlike aPRPs, they are completely cleaved by convertases during granule storage [6]. The proteoforms generated from the pre-pro-proteins are listed below:

- Products of Locus *PRB-1*: II-2, P-E (or IB-9), P-Ko, IB-6, Ps-1, Ps-2.
- Products of Locus *PRB-2*: IB-1, P-J, P-H (or IB-4), P-F (orIB-8c), IB-8a(Con 1+), IB-8a(Con 1−).
- Products of Locus *PRB-4*: PGA, II-1, Cd-IIg, P-D (or IB-5).

The nomenclature reported for bPRPs is difficult and confusing since it derives from two different criteria. Kauffmann and colleagues [10,11,12,13] have purified eleven bPRPs and determined the sequence of ten of them. They named these proteoforms using the names of the different fractions obtained from an articulate multidimensional preparative chromatographic separation of whole saliva. In the same years, Saitoh and colleagues [14,15,16] and Isemura and colleagues [17,18] identified nine bPRPs, naming them from P-A to P-I, and sequenced seven of them. As reported in the list, the two nomenclatures have several overlaps. P-A and P-I are artifacts deriving from the proteolytic cleavage occurring during the purification of salivary proteins. Although included in the class of basic PRPs, P-B and P-C peptides are not codified by *PRB1*-*PRB4* genes. Indeed, the P-B peptide is encoded by the *PROL3* gene (PBI) clustered on chromosome 4q13.317. It is related to statherin and, therefore, it cannot be considered to pertain to the bPRP family. As described in the previous section on aPRPs, the P-C peptide derives from the cleavage of four isoforms of aPRPs, namely PRP-1, PRP-2, Pif-s, and Db-s. In a top-down MS proteomic study, we were able to characterize a new bPRP deriving from the *PRB-2* locus, which was named P-J [19,20].

**Figure 3:**
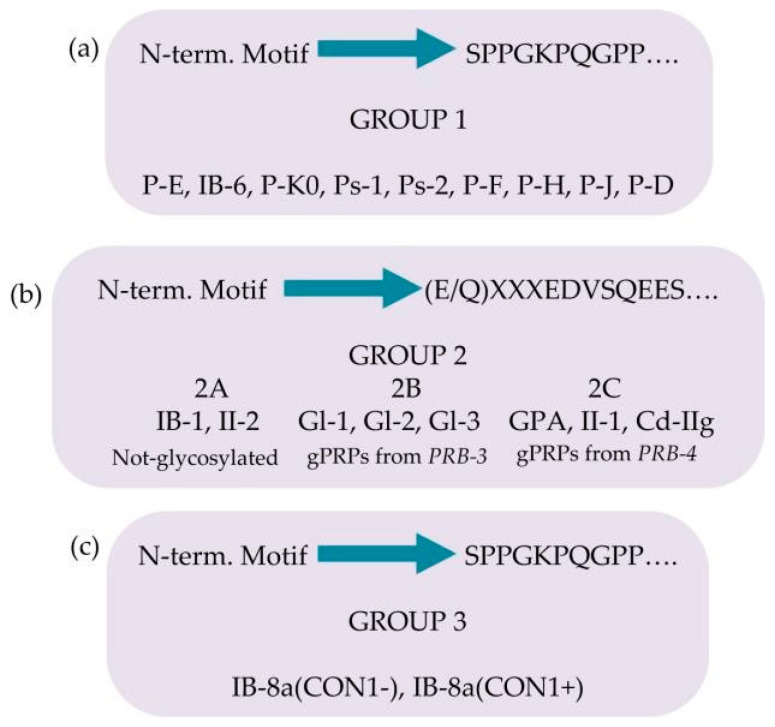
Classification of the basic proline-rich proteins (bPRPs) based on the similarity of the N-terminal motif and on the presence of glycosylations. Group 1 in panel (**a**), group 2 in panel (**b**), group 3 in panel (**c**).

The pre-secretory cleavage of bPRP pro-proteins usually occurs at the level of the consensus sequence KSRSXR↓, where X may be Pro, Ser, or Ala [6,21,22]. An extensive characterization of the different proteoforms and fragments of bPRPs has been recently published [23], where the interested reader can find the list of the bPRPs components detected until 2018. In this inventory, a new classification of bPRPs was proposed based on the similarities of their sequence, dividing them into three groups, reported in Figure 3a–c. The first group, which we named Group 1, includes P-E, P-Ko, IB-6, Ps-1, Ps-2, P-H, P-F, P-J, and P-D (Figure 3a). The sequence of all these bPRPs starts with the same SPPGKPQGPP motif, followed by sequences somewhat similar but showing small variations among the different components. The central part of the sequences shows similar repeats. Because P-E, IB-6, Ps-1, and Ps-2 sequences originate from DNA-length polymorphisms in exon 3 of the *PRB1* locus, they exhibit high similarity. The bPRP with a Mav of 10,433.5 Da, detected in whole saliva and in parotid secretory granules and named P-Ko, is encoded by *cP4*, a differentially spliced transcript of the *PRB1-L* allele.

Group 2 includes IB-1, II-2, and the glycosylated bPRPs codified by *PRB3* and *PRB4* genes, namely, Gl-1, Gl-2, Gl-3, GPA, II-1, and Cd-IIg (Figure 3b). Their sequences start with a similar motif (E/Q)XXXEDVSQEES, where XXX is LNE in IB-1, II-2, Gl-1, Gl-2, and Gl-3 and SSS in GPA, II-1, and Cd-IIg). The central part of the sequences comprises similar repeats, with differences from the repeats of the members belonging to Group 1. Based on structural differences and similarities, members of Group 2 can be divided into three subgroups: Group 2A, including IB-1 and II-2, without glycosylation sequons; Group 2B, including the Gl proteins codified by the alleles of *PRB3* locus; and Group 2C, including the glycosylated proteins codified by the alleles of *PRB4* locus. The Small Group (3) is a hybrid group, which includes the two proteoforms of IB-8a, Con1−, and Con1+ (Figure 3c). The initial sequence of these two proteins resembles that of Group 1, while the terminal sequence is like the repeat responsible for the glycosylation of the bPRPs of Groups 2B and 2C [23].

#### 2.1.2. Further Pre-Secretory Cleavages of PRPs

All the PRP families described above are substrates of a carboxypeptidase, which removes the C-terminal residue and commonly, obviously, is an arginine. However, the enzyme is not specific. It removes other residues, and sometimes a second C-terminal loss is detectable [22]. The C-terminal pre-secretory cleavages occurring on PRPs are reported in Table 1.

#### 2.1.3. Role of PRPs

Salivary proline-rich proteins are highly conserved in mammalian saliva [24]. Nonetheless, significant structural differences are present in the mammal families, suggesting that they play a relevant role in oral protection, in the modulation of the activity of oral ions, in the colonization of oral microbiota and the gastrointestinal tract, and in the feeding habit [24]. However, while aPRPs and the gPRP, called Gl 3M, are detectable in the saliva of preterm newborns, the other bPRPs are not detectable in human saliva until puberty [20,25,26], suggesting either a role in the perception of the taste of foods or a function in secondary sexual maturation. As we are aware, this information is not available for other mammals. In humans, bPRPs are secreted only by parotid glands, and this regioselectivity is also puzzling. Some bPRPs exhibit the ability to bind harmful tannins [27], others can modulate the oral flora [28,29], and some others are involved in bitter taste perception [30]. Some bPRP fragments are involved in enamel pellicle formation [31] and others act as antagonists of progesterone-induced cytosolic Ca^2+^ mobilization [32].

The intrinsic propensity of some fragments to adopt a polyproline-II helix arrangement joined to PXXP motifs was suggestive of the interaction with the SH3 domain family [33]. Interestingly, interactions were highlighted with Fyn, Hck, and c-Src SH3 domains [34], which are included in the Src kinases family, suggesting that some basic bPRPs can be involved in the signal transduction pathways modulated by these kinases. Only a small amount of data on correlations between genes of bPRPs and diseases linked to their allelic variants has been reported so far. In fact, for some of the alleles (PRB1VL, PRB2S, M, VL, and PRB3VL) the genetic sequence is not reported. Moreover, for the small and large alleles of PRB1, the genetic sequence is incomplete [35] because the reference genome (NCBI Gene ID: 5542) encodes the medium allele. Regarding the primary structure of bPRP alleles, in the UniProtKB database (accession number P04280), the full amino-acid sequence of the large variant, deduced through experimental evidence at the protein level, is deposited.

#### 2.1.4. Statherin and P-B Peptides

**Figure 4:**
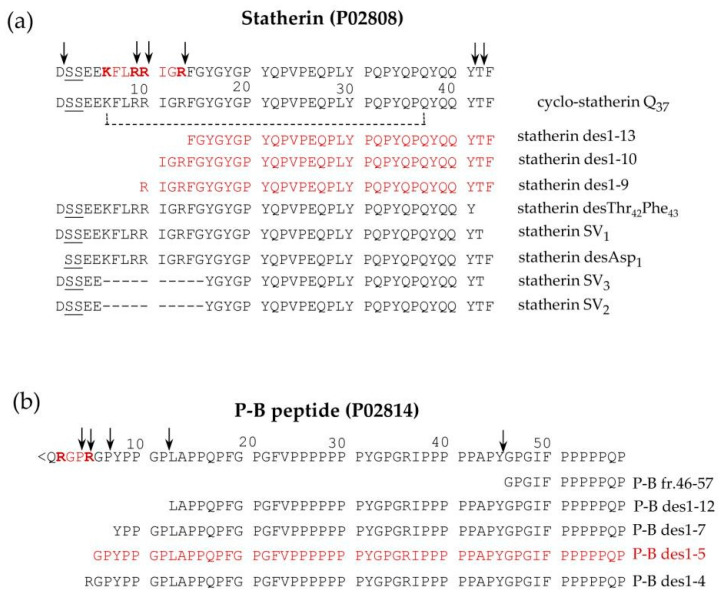
Pattern of proteoforms, mainly proteolytic fragments, originating from statherin (**a**), and the P-B peptide (**b**), characterized in human saliva by a top-down approach based on reversed-phase high pressure liquid chromatography-electrospray ionization tandem mass (RP-HPLC-ESI-MS/MS) analysis. The arrows indicate the main cleavage sites. The sequences recognized by furin-like convertase are evidenced in bold red, and the fragments derived by these cleavages are in shown in red.

Statherin is encoded by the *STATH* gene localized on chromosome 4q13.3 [36]. It is a 43-amino-acid-residues multifunctional phospho-peptide characterized by an anomalous high content of tyrosine, proline and glutamine. The roles of statherin in the oral cavity are various. Due to its great affinity for calcium phosfate minerals, such as hydroxyapatite, it maintains the supersaturated state of calcium in human saliva, thus contributing to the mineral dynamics of the tooth surface and stabilizing the acquired enamel [37]. Furthermore, statherin is involved in bacterial colonization [38] and acts as a boundary lubricant on the enamel surface [39]. P-B is the product of the specific *PROL3* gene located on chromosome 4q13.3, very close to the *STATH* gene [40]. Differently from bPRPs, which do not have aromatic amino acids in their sequences, and similarly to statherin, it has three tyrosine residues in its sequence. Furthermore, like statherin, the P-B peptide is secreted mainly by submandibular/sublingual (SM/SL) glands and also by parotids [41]. It is important to recall that P-B is also called, at the Swiss-Prot site (code P02814), “submaxillary gland androgen-regulated protein 3B precursor”, but we were not able to find any reference justifying the origin of this name. The specific role of the P-B peptide in saliva is still far from being clarified [42,43]. Nonetheless, its sequence resembles the ionic complement of statherin. Indeed, statherin has a small N-terminal negative tail followed by a long neutral domain with many proline residues, while the P-B peptide has a small N-terminal positive tail followed by a long neutral poly-proline domain. The two peptides could interact for the formation of the acquired enamel pellicle of the tooth. Different isoforms of statherin have been detected in human saliva; the variant called SV1 (Statherin variant 1) corresponds to statherin missing the C-terminal Phe residue (Statherin desPhe_43_) [41,44] (Figure 4a). The variant SV2, showing the deletion of the 6–15 internal residues with respect to statherin (Statherin des6-15), originates from alternative splicing that excludes an exon of 30 nucleotides [45]. SV3 corresponds to the SV2 variant lacking the C-terminal Phe residue. SV-1 lacking the C terminal Phe_43_ and statherin desThr_42_Phe_43_ (Figure 4a) was detected in parotid and submandibular secretory granules, suggesting that C-terminal removal occurs during the maturation of the secretory granules [6]. On the contrary, the N-terminal removal that generates statherin desAsp_1_ (Figure 4a) is probably an event occurring after granule secretion because this derivative was not detected in granules [6]. Statherin desAsp_1_ was also detected in human saliva by Vitorino and colleagues [46].

Similarly, a small amount of P-B des1–5 was found in whole saliva as well as in parotid and submandibular-sublingual saliva but not in secretory granules, suggesting that this N-terminal cleavage also occurs after secretion [6]. Some P-B and statherin naturally occurring fragments are generated from a cleavage operated by a furin-like convertase and have been characterized by a top-down approach in our previous studies [6,41]. Statherin undergoes convertase cleavages at the consensus sequence …R9R_10_IGR_13_…, generating statherin des1-9, des1-10, and des1-13 (Figure 4a), while P-B des1-5 is a convertase fragment of P-B peptide generated at the consensus sequence …R_2_GPR_5_… (Figure 4b). The P-B peptide undergoes further cleavages at the level of various chymotryptic-like sequences, generating several fragments detectable by a top-down proteomic approach [20] (Figure 4b).

#### 2.1.5. Histatins (Hst)

Histatins are a family of small peptides deriving their name from the high number of histidine residues in their sequence. It is widely accepted that all the members of this family arise from two parent peptides, named histatin 1 and histatin 3, with very similar sequences and encoded by two genes (HIS1 and HIS2) located on chromosome 4q13 [47]. Despite the very high sequence similarity, these two peptides follow completely different PTM pathways. Histatin 3 (Hst3), differently from histatin 1 (Hst1), is submitted to a sequential cleavage, generating Hst6 (Hst3 1/25) at first, then Hst5 (Hst 3 1/24) subsequently, with powerful anti-fungal activity [48], and then other fragments [49]. Their different susceptibility to cleavage derives from the presence in Hst3 of the …RGYR↓… convertase consensus sequence, which is absent in Hst1 and thus is not cleaved. Hst1 today is considered a proangiogenic factor that may contribute to oral wound healing [50,51].

Some years ago, the group of Oppenheim [48] was able to establish the sequence of 12 histatins and named them from Hst1 to Hst12. The advent of the high-throughput MS apparatus applied to proteomic studies allowed for the determination of many other Hst fragments, the majority deriving from Hst3 [49,52]. For this reason, the nomenclature of histatins fragments has been recently modified and is reported on the Swiss Prot site with the code numbers P15515 (Hst1) and P15516 (Hst3).

#### 2.1.6. Cystatins

Cystatins include type-1 cystatins (cystatins A and B), type-2 cystatins (C, D, S, SN, SA), and kininogens, or type-3 cystatins. Various biological activities have been demonstrated for these proteins, but a major role is linked to the inhibitory action exerted against cysteine proteinases. Thus, for this ability to modulate the proteolytic system, they are considered central in various diseases, including cancer [53,54,55,56]. Cystatins S, SN, SA, C, and D are encoded by loci *CST1–5* closely clustered on chromosome 20p11.21. It has been observed that S-type cystatins (S, SN, and SA), present at higher concentrations in SM/SL secretion than in parotid saliva [6], were absent or present at very low concentrations from both parotid and SM/SL secretory granules. This finding suggested that the secretion of S-type cystatins is not granule-mediated [6]. It is relevant to remark that cystatin A and B are leaderless [57]. Cystatin C was found sporadically in parotids, SM/SL, and whole saliva, but it was absent from secretory granules, whereas cystatin D was not found in any sample. Thus, it was suggested that cystatins C and D may have a different origin than the other salivary cystatins. The presence in human saliva of truncated proteoforms of cystatins has been suggested by Lupi and colleagues, who observed by HPLC-ESI-MS several masses possibly related to N-terminally truncated cystatins in the acidic soluble fraction of human saliva [58]. Indeed, a subsequent in-depth study performed both in the acidic supernatant of whole saliva and in RP-HPLC-enriched fractions, by an integrated top-down/bottom-up pipeline, characterized some truncated proteoforms of cystatins [57]. The study evidenced that not all cystatins undergo proteolytic modifications. The following truncated proteoforms for the widespread cystatin SN and its natural variant SN Pro_11_ → Leu were detected and characterized: cystatin SN des1–4, and SN des1−7, cystatin SN Pro_11_ → Leu des1–4, and SN Pro_11_ → Leu des1–7. A truncated form of cystatin SA lacking the first seven amino acids from the N-terminus (cystatin SA des1–7) was also detected. Three truncated forms of the variant cystatin D Cys_26_ → Arg have been detected in human saliva, but none for the variant Arg_26_ → Cys. For easier reading, we will utilize the name cystatin D to refer to the variant Cys_26_ → Arg. Cystatin D des1–4 and des1–8 have been characterized by a top-down Fourier-transform ion cyclotron resonance mass-spectrometry pipeline [59] and also detected in the study of Manconi and colleagues [57] A third truncated proteoform of cystatin D lacking the first five amino-acid residues with the N-terminal glutamine converted to pyroglutamic acid (pGlu-cystatin D des1–5) was also characterized in the latter study. The authors speculated that the high abundance of the latter truncated proteoform was due to the greater resistance from degradation by amino peptidases due to N-terminal pyroglutamination. Cystatin B in adult human saliva is commonly detectable as an intact proteoform, and the two fragments 1-53 and 54-98 were characterized in the saliva of human preterm newborns [60].

### 2.2. Post-Secretory Cleavages

Almost all the families of salivary proteins and peptides described above (and others) are substrates of several proteolytic enzymes present in the mouth deriving from oral flora [61,62]. Since they are myriad, reporting a complete list is impossible, and in the following, we will refer to excellent published lists. Concerning PRPs and particularly bPRPs, many recent mass-spectrometric studies allowed us to report many bPRPs fragments [23,43,46,52], with high affinity for the tooth enamel [52]. However, the observation of a recurrent XPQ C-terminal sequence in many fragments detected induced the group of Oppenheim and Helmerhorst to characterize a glutamine endopeptidase from Rhotia sp [63]. Using the synthetic substrates KPQ-pNA and GGQ-pNA, the overall K(m) values were determined to be 97 +/− 7.7 and 611 +/− 28 micromolar, respectively, confirming glutamine endoprotease activity in whole saliva and the influence of the amino acids in positions P(2) and P(3) on protease recognition. The pH optimum of KPQ-pNA hydrolysis was 7.0, and the activity was most effectively inhibited by antipain and 4-(2-aminoethyl) benzene sulfonyl fluoride. The enzyme is metal-ion-dependent and not inhibited by cysteine protease inhibitors. A systematic evaluation of enzyme activities in various exocrine and non-exocrine contributors to whole saliva revealed that the glutamine endoprotease derives from Rhotia and is localized in dental plaque [63,64].

A further protein submitted to fragmentation in the mouth is the polymeric immunoglobulin receptor (PIgR), a type-I transmembrane glycoprotein playing the main role in the adaptive immune response on mucosal surfaces [65,66]. It transports polymeric IgA across mucosal epithelial cells. A proteolytic cleavage occurring in the glycosylated extra-cellular portion of pIgR generates the secretory component (19–603 residues), which has also been detected in human saliva [67]. The cleavage occurs by the action of unknown proteases, probably released by activated neutrophils [66], and the highly conserved sequence 602–613 (PRLFAEEKAVAD) is believed to be the cleavage signal [65]. Two peptides deriving from PIgR are detectable in saliva by a top-down proteomic approach; they are named AVAD and ASVD [68]. The peptide named AVAD originates by the cleavage occurring in this region at the level of Lys_609_, and the ASVD peptide derives from AVAD by the trypsin-like cleavage at Arg_622_. AVAD and ASVD peptides do not derive from the secretory component and have a sequence partially overlapped by the transmembrane portion (639–661) of PIgR. Thus, they should originate by degradation of PIgR after its release from disrupted cell membranes.

### 2.3. Proteolytic Cleavages: Variations Related to Age and Pathologies

Interestingly, it was observed that several proteolytic cleavages change according to age and in several diseases. For instance, as previously reported in Section 2.1.6, Iavarone and colleagues [60] detected in the saliva of human preterm newborns sensible amounts of cystatin B fragments 1–53 and 54–98, suggesting in foetuses the presence of high-active specific proteolytic events that disappeared in adults. The cleavage involves a tyrosine and phenylalanine couple (Tyr↓Phe) and can be ascribed to a chymotrypsin-like enzymatic activity with strict specificity, as suggested by the very precise consensus sequence necessary for the cleavage. The two fragments were detected at very high relative amounts with respect to intact cystatin B in very preterm newborns, for which the concentration decreased as a function of the post-conceptional age (PCA) and they were practically undetectable when the age corresponded to that of full-term newborns. A search on the Merops protease database (release 12.4, accessed on 9 January 2017, http://merops.sanger.ac.uk/) returned various possibilities other than chymotrypsin A, such as chymosin, cathepsin E, metalloproteinase 2, ADAMTS4, endothelin converting enzyme 1, and some peptidases of the chymase class (mast cell chymotrypsin-like proteinase). It is impossible to establish if cystatin B is a natural substrate of the enzyme, thereby implying a functional role for the fragments as potential cryptides, or if the fragments observed are byproducts of a proteinase, without any functional meaning, their activity is devoted to other specific foetal oral cleavage processes. Similarly, an increased activity of convertases and carboxypeptidases responsible for the cleavages of aPRPs, histatins, and statherin was observed in preterm newborns with a low PCA of approximately 190 days. The activity decreased according to the PCA, and it reached the levels observed in the adult around the normal term of delivery [69]. Interestingly, this behavior was observed for the release of the PRP3-type aPRPs and the P-C peptide from PRP-1-type aPRPs, as well as for the release of histatin 6 and histatin 5 from histatin 3 and for the release of the fragments of statherin missing C-terminal residues. This was not the case for statherin desAsp_1_, which showed low levels or was not detectable in preterm newborns at low PCA but increased after birth, reaching values similar to those determined in at-term newborns [70]. Because statherin desAsp_1_ is a fragment of statherin not detectable in granule preparations [6], this result is a further clue that the proteinases (convertases and carboxypeptidases) involved in the cleavages of these families of salivary proteins are confined in the Golgi apparatus.

It has been reported that proteolytic fragmentations of salivary proteins may vary in some pathological states compared to physiological conditions. For instance, a study performed on a group of children and adolescents affected by type-1 diabetes revealed that several small peptides, most likely originated by post-secretory proteolytic cleavages occurring in the mouth, showed a higher concentration with respect to sex- and age-matched controls, suggesting increased activity of exogenous proteinases in the oral cavity of diabetics [70]. The peptides, characterized as fragments 1–14, 1–25, 5–25, 26–35, 26–44, and 36–44 of the P-C peptide, have been already detected in saliva from healthy subjects [63], and fragments 1–14 and 26–44 were also detected in the parotid saliva of healthy subjects by Hardt and colleagues [71]. Furthermore, an association of fragments 1–14 and 26–44 to high numbers of dental caries and the presence of fragments 1–14 in the saliva of subjects affected by Sjogren’s syndrome have been demonstrated by Huq and colleagues [72]. A top-down proteomic study investigated the salivary proteome of 49 multiple sclerosis (MuSc) patients and 54 healthy controls, quantifying 119 salivary peptides/proteins [73]. Among the observed differences, the fragments 1–14, 26–44, and 36–44 of the P-C peptide, the SV1 fragment of statherin, and cystatin SN des1–4 showed higher levels in patients with respect to controls. In a study performed to characterize possible differences in the salivary proteome of subjects affected by Wilson’s disease (WD) with respect to healthy controls, increased levels of the AVAD and ASVD fragments of PIgR (see above) were observed in patients, probably because of an increased disruption of cell membranes due to the high production of ROS typical of WD [68]. A higher level of the ASVD peptide was also determined in MuSc patients with respect to healthy controls [73]. Interestingly, a study performed on patients affected by systemic mastocytosis (SM) and grouped into SM with (SM+C) or without (SM-C) additional cutaneous lesions evidenced that the two SM forms were distinguished by the lower levels of PRP-3, PRP-3 desArg_106_, statherin desPhe_43_, P-B des1–5, and cystatin D des1–5 and des1–8 in SM-C patients with respect to SM+C [74]. It should be outlined that lower levels of cystatin D des1–5 and des1–8 have been also observed by comparing saliva of healthy elderly subjects with respect to adults [75]. 

The study was performed on patients affected by autoimmune hepatitis (AIH) and primary biliary cholangitis (PBC), two autoimmune liver diseases characterized by chronic hepatic inflammation and progressive liver fibrosis, to establish a panel of salivary proteins/peptides able to classify with good accuracy PBC patients vs HCs, AIH patients vs HCs, and PBC vs AIH patients. Among the other data, they revealed significantly different levels of PRP-3 desArg_106_ by comparing the two patient groups [76].

Higher levels of statherin desPhe_43_ and two others naturally occurring fragments (des1–9, and des1–13) were measured in the saliva of a group of Alzheimer’s disease patients compared to controls [77].

## 3. Phosphorylation

Phosphorylation is probably (after proteolytic cleavage) the most-common PTM in human saliva. Generally, phosphorylation is a reversible PTM, catalyzed by more than 500 different human protein kinases, while de-phosphorylation is due to enzymes called phosfatases [78]. The detection of phosphorylated proteoforms in human saliva is strongly dependent on the proteomic pipeline utilized: bottom-up or top-down. Commonly, the bottom-up strategies are accomplished by enrichment capture in order to increase the number of phosphorylated fragments.

A study of the group of Oppenheim and Helmerhorst [79] utilized chemical derivatization using dithiothreitol (DTT) of the phospho-serine/threonine-containing peptides obtained after the trypsin digestion of whole-saliva samples. The DTT-phospho-peptides were enriched by covalent disulfide-thiol interchange chromatography and analysis by nanoflow liquid chromatography and electrospray ionization tandem mass spectrometry (LC-ESI-MS/MS). The specificity of DTT chemical derivatization was evaluated separately under different base-catalyzed conditions with NaOH and Ba(OH)_2_, blocking cysteine residues by iodoacetamide and enzymatic O-deglycosylation prior to the DTT reaction. Further analysis of whole-saliva samples that were subjected to either of these conditions provided supporting evidence for phosphoprotein identifications. The combined chemical strategies and mass-spectrometric analyses identified 65 phosphoproteins in whole saliva; of these, 28 were based on 2 or more peptide identification criteria with high confidence and 37 were based on a single phospho-peptide identification. Most of the identified proteins (∼80%) were previously unknown phosphoprotein components.

A phospho-proteomic study based on a bottom-up pipeline on human saliva also generated a large-scale catalog of phosphorylated protein fragments [80]. To circumvent the wide dynamic range of phosphoprotein abundance in whole saliva, the proteomic platform combined dynamic range compression using hexapeptide beads, strong cation exchange HPLC peptide fractionation, and immobilized metal-affinity chromatography prior to mass spectrometry. In total, 217 unique phospho-peptides sites were identified representing 85 distinct phosphoproteins at 2.3% global FDR. From these peptides, 129 distinct phosphorylation sites were identified, of which only 57 were previously known. Cellular localization analysis revealed salivary phosphoproteins had a distribution like all known salivary proteins but with less relative representation in “extracellular” and “plasma membrane” categories compared to salivary glycoproteins. Sequence alignment showed that phosphorylation is mainly linked to the action of the Golgi casein kinase called Fam20c (see below), but it also occurred at acidic-directed kinase, proline-directed, and basophilic motifs [80].

The top-down pipelines are more conservative, giving precise information on the salivary protein substrate of kinases and on their sites of modification. All the phosphorylated proteins pertaining to the secretory pathway are phosphorylated by a pleiotropic Golgi casein kinase which, until a few years ago, was elusive. An interesting study performed by Tagliabracci and colleagues [81,82] was able to establish that the enzyme is the kinase named Fam20C. The main consensus sequence of Fam20C is a serine with a +2 specific negative residue, either glutamic acid or phospho-serine (SXE/S(phos)). The salivary proteins and peptides submitted to the action of Fam20C in agreement with this consensus sequence are reported in Table 2.

The phosphorylation of cystatin S, whose N-terminal sequence is S_1_SS_3_KE_5_…, is interesting. Monophosphorylated cystatin S is called cystatin S1, and due to the presence of the glutamic acid residue in position +2, Ser_3_ is necessarily the first site of phosphorylation. Di-phosphorylated cystatin S is called cystatin S2, and the second phosphorylation at Ser1 is strictly hierarchical, occurring only after that of Ser3. Cystatin S1 is the most abundant in adult human saliva and the approximate ratio between the relative percentages of the three components (S, S1, and S2) is 5:80:15, respectively [58,83]. A more complex situation concerns the phosphorylation of aPRPs, which are commonly di-phosphorylated, having Ser_8_ and Ser_22_ as the two main sites of phosphorylation [9,84]. However, by mass spectrometry, it is possible to detect also in small amounts non-, mono-, and three-phosphorylated aPRPs (on Ser_8_, Ser_22,_ and Ser_17_) [9] (Figure 2). The consensus sequence recognized for Ser_8_ phosphorylation is the canonical S_8_QE, with a negatively charged residue at position +2, while the phosphorylation of Ser_22_ by Fam20c is ensured by the recognition of the secondary consensus sequence S(X)_3-4_ (D/E/S(phos))_3_ [84]. To emphasize that the phosphorylation of Ser_22_ allows for the hierarchical phosphorylation of Ser_17_, which is located in the sequence …S_17_DGGDS_22_EQFIDEE… [9]. Hence, the mono-phosphorylated aPRPs can be phosphorylated either on Ser_8_ or on Ser_22_ (with a different proportion in favor of Ser_8_) [9]. The di- phosphorylated components (the most abundant) are commonly phosphorylated on Ser_8_ and Ser_22_, but a very small percentage of aPRPs phosphorylated on Ser_17_ and Ser_22_ could be present. The hierarchical phosphorylation on Ser_17_ is further substantiated by a study characterizing the aPRP-1 Roma–Boston Ser_22_(phos) → Phe variant, which was never detected as di-phosphorylated proteoform, also using high-resolution HPLC-MS apparatus [85]. Statherin is mainly di-phosphorylated on Ser_2_ and Ser_3_ but with the N-terminal sequence being DS_2_S_3_EE…, the two phosphorylation sites are independent and the phosphorylated Ser of the mono-phosphorylated proteoform, always detectable in adult human saliva, can be either Ser_2_ or Ser_3_. The N-terminal sequence of Hst1 is DS_2_HE…, and about 90% of the peptide is phosphorylated on Ser_2_, and 10% of the non-phosphorylated peptide is detectable in adult human saliva [49]. Even though the Fam20C consensus sequence was not respected, a study by Halgand [86] and colleagues, carried out with a top-down pipeline, evidenced a second minor phosphorylation site on Ser_20_.

The canonical consensus sequence of Fam20C is also responsible for the phosphorylation of several bPRPs. Indeed, the IB-1 and II-2 proteoforms are both phosphorylated on Ser_8_. Among the gPRPs Gl-1, Gl-2, Gl-3, GPA, II-1, and Cd-IIg, which have an N-terminal sequence motif similar to IB-1 and II-2, ((E/Q)XXXEDVS_8_QEES…, where XXX is LNE in IB-1, II-2, Gl-1, Gl-2, and Gl-3 and SSS in GPA, II-1, and Cd-IIg), Gl-2 is phosphorylated on Ser_8_ [23]. Phosphorylation is an almost complete event because <1% of the non-phosphorylated forms can be detected in parotid granules, parotids, and whole saliva and probably occurs after the cleavage of the proprotein [6]. It can be supposed, by sequence similarity, that Gl-1 and Gl-3 also undergo the same PTMs, although experimental evidence is missing. The presence of the …S8QE consensus sequence of GPA, II-1, and Cd-IIg (Group 2C of bPRPs) also suggests the phosphorylation of Ser_8_ for these bPRPs, even though these modifications have not been experimentally evidenced until now. A second potential, but not demonstrated, phosphorylation site at Ser_3_ is present in the sequence of Group 2C bPRPs (<ESS_3_SED…). The activity of other kinases can be revealed in human saliva by using a top-down proteomic approach, among them MAPK14, a kinase pertaining to the p38 mitogen-activated protein kinase pathway that can partly phosphorylate the protein S100A9 on the penultimate Thr residue of its sequence [87].

### Variation of Phosphorylation as a Function of Age and for the Diagnosis of Different Disease

Some bottom-up studies suggested that the analysis of the phospho-proteome of salivary extracellular vesicles could offer a possibility for either the diagnosis of lung cancer [88] or to distinguish oral squamous cell carcinoma patients from healthy individuals [89]. Our studies were able to evidence that during the last months of foetal growth, the phosphorylation of secretory salivary proteins, and therefore the activity of Fam20C, is very low, if not completely absent [90]. It increases slowly, reaching the level observed in adults a few weeks after the normal time of delivery [25,91]. Interestingly, a top-down study of the salivary proteome performed in a group of children with autism spectrum disorders evidenced significantly lower phosphorylation levels of four salivary peptides, in comparison with age- and gender-matched healthy controls, providing a clue as to the molecular pathogenesis responsible for these disorders [92].

## 4. Sulfation

Sulfonation (or sulfation) is usually an irreversible PTM. It consists of the transfer of a sulfate group (-SO_3_^−1^) from the only known sulfate donor, i.e., 3′-phosphoadenosine-5′-phospho-sulfate (PAPS), to endogenous substances such as proteins, carbohydrates, and catecholamines as well as estrogenic steroids and xenobiotics [93]. Moreover, 3′-phosphoadenosine-5′-phosphosulfate synthase (PAPSS) is the enzyme responsible for biosynthesizing PAPS during two reactions: inorganic sulfate is first converted to adenosine-5-phosphosulfate (APS) by ATP sulfurylase (EC 2.7.7.4), and this intermediate molecule is then phosphorylated by the APS kinase (EC 2.7.1.25) to form PAPS [94]. Both APS and PAPS are activated sulphuryl donors that possess a phospho-sulfate anhydride bond [95]. In prokaryotes, fungi, and plants, the synthesis of PAPS is performed by two separate enzymes [94]. In the animal kingdom, however, the ATP sulfurylase and the APS kinase are encoded by the same gene and translated into a single polypeptide that forms the dual-functional enzyme PAPSS [95]. PAPSS1 and PAPSS2 are two characterized isoforms of this enzyme, according to their different localization. Moreover, a relation of various pathological conditions to deficiencies of PAPSS (both isoforms) has been demonstrated [96]. While PAPSS1 and PAPSS2 are responsible for the bioactivation of sulfate, sulfo-conjugation reactions are catalyzed by enzymes known as sulfotransferases [97]. Sulfotransferases are mainly divided into two groups, as they are either cytosolic or membrane-bound [98]. Cytosolic sulfotransferases constitute the superfamily of enzymes known as SULTs, which are involved in the sulfonation of xenobiotics and small endogenous compounds such as neurotransmitters and hormones [99]. The membrane-bound sulfotransferases are found in the Golgi apparatus and are responsible for the post-translational sulfation of endogenous macromolecules such as proteins, lipids, and glycosaminoglycans [100]. Currently, 12 SULT isoforms have been identified and detected in human tissues [101]. The availability of PAPS in different tissues can highly affect the sulfation pathway by modifying the affinity of sulfotransferases [100,101,102].

In human saliva, we were able to determine that Hst1 is partly sulfated on the last four tyrosines (out of five) of its sequence [103], and until now, this was the only phospho-sulfo-peptide detected in human saliva. As previously reported, the phosphorylation of Hst1 is not a complete event, because in whole human saliva, it is possible to detect about 10% of the non-phosphorylated peptide. Further studies performed by high-resolution HPLC- MS apparatus suggested that: (a) as supposed in our work, the sulfation process is hierarchical, with Tyr_27_ being the first residue sulfated, followed by Tyr_30_, Tyr_34,_ and Tyr_36_. Indeed, MS CID fragmentation data on the mono-sulfated (non-phosphorylated) Hst1 clearly indicated Tyr_27_ as the unique residue involved; (b) the sulfation process is strictly confined to the submandibular gland, because the sublingual gland does not express Hst1, and Hst1 secreted by the parotid gland is not sulfated; (c) the phosphorylation and the sulfation processes are independent, because, by the study of the neutral losses generated during the MS/MS CID-induced fragmentation, it is possible to discriminate non-phosphorylated poly-sulfated derivatives from the phosphorylated ones; (d) the percentages of the different polysulfated derivatives vary sensibly in the human submandibular saliva of humans; (e) the sulfation of Hst1 is not detectable in children until puberty; and (f) potential changes in physio-pathological conditions and during pharmacological treatments require extensive statistical analysis in a large population.

## 5. S-Modifications

**Figure 5:**
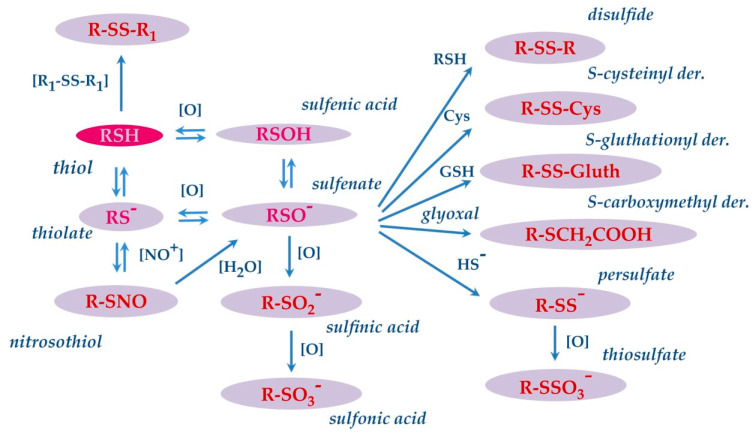
Map of reversible and irreversible oxidative modifications that can occur on the thiol group of the Cys residue.

The thiol side chain of cysteine residues present in the proteins is characterized by high redox sensitivity. This redox sensitivity derives from the pKa of the thiol group, which due to the environment of the protein, even at physiological pH, may be partly present in the thiolate form (RS^−^) and more reactive than the thiol form (RSH). Indeed, RS^−^ is prone to donating an electron pair to target reagents, generating the oxidized derivative sulfenic acid (RSOH), mainly present as sulfenate (RSO^−^), by a reversible reaction. Then, sulfenate may undergo other reversible and irreversible oxidative modifications (Figure 5) [104]. For instance, it can rapidly react with another thiols (RSH) by generating the corresponding disulfide (RSSR), or with GSH or free Cys to form S-glutathionyl (RSSG) or S-cysteinyl derivatives. This is not the only formation pathway for disulfide derivatives, since the latter can also be generated in a process termed thiol-disulfide exchange, in which a thiol form reacts with a disulfide to generate a different thiol and disulfide.

In the presence of strong oxidants, sulfenate can undergo further oxidation and generate sulfinic (RSO_2_H) and sulfonic acid (RSO_3_H) derivatives sequentially. The latter two modifications are regarded as irreversible and are associated with oxidative damage. Sulfenate can also react with hydrogen sulfide (HS^−^) to form a cysteinyl persulfide (R–SS^−^), which can be oxidized to cysteinyl thiosulfate(R–SSO_3_^−^). The thiol side chain of cysteine may also react with nitric oxide, resulting in the formation of nitrosothiol derivatives [105]. These modifications have been observed and characterized on specific salivary proteins as cystatins and S100A proteins. Cabras and colleagues evidenced that in human whole saliva, cystatin B is present mostly as S-modified derivatives on Cys_3_, being the S-unmodified proteoform rarely detectable in the saliva of healthy adults. Generally, more than half of cystatin B (55%) is found to be S-glutathionylated, 30% is present in the dimeric form, and the remaining 15% is S-cysteinylated [106]. In an in-depth study, performed both in the acidic supernatant of whole saliva and in enriched fractions obtained by preparative RP-HPLC, the structures of all these S-modified proteoforms of cystatins were confirmed by an integrated top-down/bottom-up proteomic approach [57]. In the study, a carboxymethylated Cys_3_ derivative of cystatin B was also detected and characterized. Carboxymethylation, like the formation of sulfinic and sulfonic acid derivatives, is a nonenzymatic, irreversible, and stable modification generated by the endogenously formed glyoxal with Cys sulfhydryl groups (Figure 5). This modification was novel not only for cystatin B but also for any other human salivary protein. It should be outlined that Cys residue present in the N-terminal region at the C3 position of cystatin B is involved in the formation of these and other derivatives. The N-terminal region of cystatin B is crucial for the biological function of the protein, being involved in the binding of cysteine proteinases [107]. Indeed, it has been shown that Cys_3_ is the most important residue for the interaction with papain and cathepsin H and is also a major contributor to cathepsin-L binding [108]. Differently from adults, the S-unmodified cystatin B represented the main proteoform in the saliva of preterm newborns. Interestingly, it was observed that the high relative amount the unmodified cystatin B observed in preterm newborns decreased as a function of the PCA, reaching values like those determined in at-term newborns, children, and adults at the normal term of delivery [60]. As reported in Section 2.1.6 when describing the proteolytic cleavages, in the same study, high relative amounts of fragments 1−53 and 54−98 of cystatin B were detected in very-preterm newborns. The N-terminal 1–53 fragment was detected both unmodified and modified at the level of Cys_3_ residue, with the percentage of the different forms being like those determined for the intact protein [60].

S100 proteins constitute the largest family of calcium-binding proteins, with more than 3000 related entries in the NCBI Reference Sequences Data Bank. They are EF-hand calcium-binding proteins and bind calcium via helix–loop–helix motifs often present in multiple copies. The “S100” name originates from the solubility of the first identified S100 proteins in 100% ammonium sulfate solution. The members of the EF-hand superfamily can be divided according to their calcium affinity and their ability to change conformation following the binding of calcium. Intracellular functions of S100 proteins include: (i) regulation of protein phosphorylation by interaction with the substrates of the kinases, thus playing a role in signal transduction; and (ii) regulation of enzyme activity [109].

In the extracellular milieu, S100 proteins do not function as Ca^2+^ sensors as they are saturated by the mM range Ca^2+^ concentration; however, they are recognized as playing an important role in mediating inflammatory responses through the activation of several cell surface receptors, after their release from activated or necrotic cells [110]. S100A8, S100A9, and S100A12 are constitutively expressed in high amounts in neutrophils and are inducible in macrophages (cells that generate high amounts of ROS), while the expression of the complex S100A8/S100A9 (calprotectin), S100A12, and S100A7 can be induced in keratinocytes, endothelial cells, and epithelial cells during inflammation [111].

Human S100A9 is encoded by a single copy gene with two isoforms—full-length and -truncated S100A9—and is translated from an alternate start site at codon 4 of the full-length form and lacks the single Cys_3_ residue, making it less susceptible to oxidation. In the studies of Lim and colleagues [112,113] the many pro-inflammatory functions described for S100A8 and S100A9, as well as their anti-inflammatory roles in wound healing and protection against excessive oxidative tissue damage, are discussed, and an explanation that oxidative modifications may act as a regulatory switch for the disparate functional roles of S100A8 and S100A9 is suggested.

Concerning biofluids, in a series of studies carried out to investigate possible salivary biomarkers of pathologies both confined to the oral cavity and those systemic in nature, different oxidized derivatives of S100A8 and S100A9 proteins were revealed and characterized both in healthy and diseased subjects. The structure of these new proteoforms was established by high-resolution HPLC-ESI-MS/MS analysis of the mixture of peptides, obtained by trypsin digestion of salivary fractions enriched with S100A8 and S100A9 oxidized derivatives [68]. In this study, the long form of S100A9 was detected as being glutathionylated at Cys_3_ in 15/32 subjects and cysteinylated only in 3/32, while S100A8 was sporadically detected as unmodified S100A8 (4/32), and as a sulfonic derivative at Cys_42_ (5/32).

Glutathionylated and cysteinylated S100A9 derivatives were also detected in the saliva of human preterm newborns [114]. Finally, the nitrosylated derivative of S100A8 was also observed in saliva from adult healthy subjects, but sporadically [73,74].

### S-Modifications of Salivary Proteins in Pathologies

A wider variety and abundance of S-modified proteoforms have been revealed in the saliva of subjects affected by various pathologies. For instance, a study performed on saliva collected from patients with schizophrenia and bipolar disorder, compared to healthy non-smokers and smokers control groups, revealed a more than 10-fold increase in salivary levels of S-cysteinylated and S-glutathionylated cystatin B, in addition to α-defensins 1–4, S100A12, and cystatin A, suggesting the dysregulation of the peripheral white-blood-cell immune pathway associated with the pathologies [115]. S-glutathionylated cystatin B was also found at high levels, predominantly in antibody deficiencies [116]. In WD patients, top-down proteomic analysis of saliva revealed significantly higher levels of S100A9 and S100A8 and some of its oxidized proteoforms, with respect to controls [68]. S100A8 oxidizing at Cys_42_ to sulfinic acid (S100A8-SO_2_H) was only detected in patients, while the sulfonic derivative (S100A8-SO_3_H) oxidizing at Trp_54_ was detected both in patients and healthy controls, even if with different levels. The latter proteoforms were subjected to further oxidation to give the so-called hyperoxidized S100A8, with the second oxidation located at either Met_1_ or Met_78_ (position to be determined). S100A8 was also shown to undergo glutathionylation, nitrosylation, and the formation of a disulfide bridge with the Cys_3_ of long S100A9 (S100A8/A9-SS dimer), but only in the patient group. In the same group, for the first time, the homodimer proteoform of long S100A9 was characterized. Overall, the salivary proteome of WD patients reflected the oxidative stress and inflammatory conditions characteristic of the pathology, highlighting differences that could be useful clues of disease exacerbation [68].

Oxidative cross-linking via disulfide bonds of S100A9 and S100A8 has also been observed by Hoskin et al. in saliva from healthy adults and in lavage fluid from the lungs of patients with respiratory diseases [117]. The authors showed that reactive halogen species promote the cross-linking of the non-covalent heterodimer of S100A8/S100A9 and hypothesized that the cross-linking detected in the saliva samples was most likely mediated by hypothiocyanous acid produced by lactoperoxidase. They also demonstrated that the formation of the disulfide cross-linked derivative enhanced susceptibility to proteolysis by neutrophil proteases. Furthermore, Gomes et al. observed that sulfinic and sulfonic acid derivatives of monomeric S100A8, together with novel oxathiazolidine oxide/dioxide forms, were present in asthmatic sputum [118].

The salivary proteome of Alzheimer’s disease (AD) patients also highlighted elevated levels of some S100A8 and S100A9 oxidized proteoforms with respect to age- and gender-matched healthy controls. They were the hyperoxidized proteoforms of S100A8, S100A8-SNO, and glutathionylated long S100A9 [77]. This finding was not surprising since oxidative modifications of proteins are common in neurological disorders, due to the strongly oxidizing characteristics of the extracellular milieu because of the generation of ROS and re-active nitric oxide species [119]. Higher levels of S100A8-SNO were also determined in MuSc patients with respect to healthy controls, while levels of mono- and di-oxidized cystatin SN, mono- and di-oxidized cystatin S1, and mono-oxidized cystatin SA were lower [73]. The reduced level in the patients of oxidized derivatives of S-type cystatins was an intriguing result. Indeed, high levels of oxidative stress markers and lower antioxidant status have been reported in the saliva and plasma of MuSc patients under corticosteroid therapy by Karlik and colleagues [120]. However, different results were obtained by Schipper and colleagues [121], which highlighted a positive effect of immunotherapy on oxidative stress, evidenced by a reduction of oxidative stress markers in MuSc patients. Manconi et al. speculated that reduced levels of cystatin oxidation in MuSc patients could be related to the treatment of 32/49 patients enrolled in the study. Indeed, S100A8-SNO was found at a significantly higher level only in the group of untreated patients with respect to controls [73].

Recently, analysis of the salivary proteome of patients affected by AD compared to healthy adult and elderly controls has highlighted significantly higher levels of S100A8-SNO and hyperoxidized S100A8, as well as glutathionylated S100A9 (long), in healthy adults with respect to the elderly, and in AD patients with respect to healthy elderly individuals. The same trend was observed for both glutathionylated and dimeric cystatin B, but not for the cysteinylated proteoform, which showed a significant difference only by comparing patients and elderly subjects [75].

## 6. Transglutamination

Several salivary proteins are involved in the formation of proteins layers, i.e., the so-called “oral pellicles” The general term for these layers is pellicles, but due to the different characteristics of the coated surfaces, the enamel pellicle and mucosal pellicle are their own entities. These protein films have a dual role because one, “the acquired enamel,” is important for the integrity of the tooth, and the second, the “mucosal pellicle,” is important for the protection of the oral mucosa. There is considerable information on the enamel pellicle: many proteins and their fragments are involved with a particular concern for PRPs, statherin, histatins, and the P-B peptide [122,123]. On the contrary, only limited data are available on the mucosal pellicle [124]. This can be attributed to the difficulty to develop a standardized preparation of this latter biological structure. However, it has a completely different ultrastructure as compared with the enamel pellicle. Since it is comprised of larger glycoproteins retaining water, it might be considered a hydrogel, and it appears to have a lower tenacity than the enamel pellicle. Maturation and turnover are influenced by the delivery of salivary proteins, the flow of saliva, and the underlying desquamating oral epithelium. Its probable functions include lubrication and moisture retention. Furthermore, interactions between mucosal pellicle proteins and bacterial surfaces are responsible for the specificity of the bacterial colonization during the earliest stage of plaque formation [125]. The in vivo pellicle is thought to be an insoluble network of proteins generated by the post-secretory processing of proteins, mainly due to cross-linking. Cross-links for the formation of oral mucosal pellicles were demonstrated firstly by Bradway and colleagues, which highlighted the existence of a network of proteins formed by components of saliva adsorbed onto buccal epithelial-cell surfaces that cover the oral mucosal surface [126]. Recently, a new proteomic protocol was optimized to investigate the proteins participating in the composition of the oral mucosal pellicle, and among them, the proteins of the PLUNC family were identified [127].

The oral mucosal pellicle is a thin lubricating layer generated by the binding of saliva proteins on epithelial oral cells [128]. This protein molecular network interacts with the oral epithelial-cell plasma membrane and its associated cytoskeleton and contributes to mucosal epithelial flexibility and turnover. It was demonstrated that acidic-proline-rich proteins, statherin, the major histatins, and mucins are substrates of oral transglutaminase 2 (TG2) and participate in cross-linking reactions [126] as putative pellicle precursor proteins. Whatever the structure of these protein networks may be, oral transglutaminases (mainly type-2 transglutaminase) are the pivotal enzymes for pellicle formation. TG2 can cross-link acidic PRP-1 and statherin in vitro [122]. TG2 is the ubiquitous tissue enzyme also expressed in oral epithelial cells [129], which catalyzes different biological processes and generates a cross-link between two peptide chains, typically between the ε-amine of the lysine residue (acting as the lone-pair donor) and a glutamine residue (the lone-pair acceptor), and the reaction is accomplished by the loss of an ammonia molecule. TG2 is a Ca^2+^-dependent enzyme released by the epithelial oral cells; it is negatively modulated by GTP [129] and affected by the reversible formation of an intramolecular disulfide bridge [130]. Recently, a study of our group [131] showed that also bPRPs and the P-C peptide are potential substrates of TG2. Nonetheless, they showed a very different reactivity for monodansyl-cadaverine (used as a lone pair donor). Mass-spectrometry analyses of the reaction products highlighted that P-C, P-H, and P-D (both Pro_32_ and Ala_32_ variants) peptides were active substrates of TG2; II-2 was less reactive, while P-F and P-J peptides showed negligible activity [131]. MS characterization suggested that the consensus sequence for the linking is connected more to the environment of glutamine residue than to the donor ammine. The pivotal residues characterized for P-H, II-2, and both variants of P-D evidenced …GNPQ… as the consensus sequence recognized by TG2 on bPRP peptides [131]. This consensus sequence is not present in P-F and P-J peptides and, probably for this reason, they were poor substrates for TG2.

P-C, P-H, and P-D peptides formed cyclo-derivatives after the TG2 reaction, and only specific glutamine residues were involved in the cycle formation and reacted with specific monodansyl-cadaverines [131]. The stereospecificity of TG2 was at first recognized on statherin, which under the action of TG2 forms a cyclic derivative involving almost only Gln_37_ (out of seven glutamine residues) and Lys_6_ (as a unique lysine residue) in the sequence (Figure 4) [132]. The detection of a small amount of cyclo-statherin in adult-human whole saliva [132] and the high reactivity of secondary glutamine residues after the formation of cyclo-statherin and cyclo-P-C were suggestive of the in vivo formation of ring structures with a pivotal role in the architecture of the oral mucosal and enamel pellicles [133,134]. Whatever the molecular mechanism towards the structure of the oral pellicles, they have a relevant role in the protection of the mucosa due to the mechanical and thermal high stresses that the mouth undergoes during the human life.

## 7. Glycosylation

### 7.1. Non-Enzymatic Glycosylation (Glycation)

Non-enzymatic glycosylation or glycation is a ubiquitous process involving proteins, peptides, lipids, and nucleic acids. It consists of a nucleophilic addition of sugar (commonly glucose) to the free amino group of a biological molecule (in protein, typically the ε-amino group of lysine). The first studies regarding glycation processes were carried out by the food industry for the quality control of foodstuffs [135], and only forty years later, this process was observed in the human body for monitoring the trend of glycated hemoglobin in diabetic patients [136,137,138]. The glycation process starts with the formation of a reversible but unstable Shiff’s base, followed by intermolecular rearrangement into stable Amadori products. When large amounts of Amadori products are formed, they rearrange to form a heterogeneous group of protein-bound moieties, termed advanced glycated end products (AGEs) [139]. Fluorescent spectroscopy is usually used to monitor the rate of formation of AGEs, considering that most of the advanced products of glycation are fluorophores [140]. The rate of these reactions is quite slow, and only proteins with large amounts of lysine residues undergo glycation, with significant amounts of AGEs leading to reduced protein functionality and stability [141,142].

Proteins with a long metabolic turnover, as extracellular matrix proteins, are the most susceptible to non-enzymatic glycosylation, compared to most proteins that counteract the impact of glycation thanks to their high turnover rate and short half-life [143]. For instance, the fast hematic turnover of serum albumin offers the chance to monitor short-term diabetes [144]. It is known that proteins from saliva show a very quick turnover; nevertheless, few studies point out how the loss of functional activity of some salivary proteins is to be related to glycation processes. The antibacterial activity of lactoferrin or lysozyme and the antioxidant capacity of superoxide dismutase, all detectable in human saliva, decreased after the formation of AGEs [142,145,146].

AGEs accumulate in the human body by binding to AGE-specific receptors (RAGEs), which play an important role in the development of age-related organ hypofunction [139]. The presence of RAGEs was also evidenced in the oral cavity, particularly in the minor salivary glands [147]. Manig and colleagues, in 2019 [148], carried out the first study focused on monitoring the free salivary glycated compounds predominant during later reaction stages of AGEs production in saliva. Specifically, by LC-MS-MS analysis, the concentration of Nε-carboxymethyllysine (CML), pirralyne (Pyr), methylglyoxal-derived hydroimidazolone 1 (MG-H 1), and Nε-carboxyethyllysine (CEL) was evaluated [148]. The presence of AGEs in saliva increases the risk of developing periodontitis and pulpitis [147]. Furthermore, the expression of RAGEs appeared to be correlated with the histologic differentiation of oral squamous cell carcinomas [149]. Maciejczyk M. and colleagues [150] evidenced the role of saliva as a diagnostic biofluid alternative to blood for assessing the severity of protein glycation, correlated to the aging processes. Several proteomic studies, mainly performed by top-down mass-spectrometry approaches, have shown that saliva can be a mirror of the aging process. Indeed, age-related changes in salivary proteome profiles have been observed in individuals from 180 days to the elderly [75]. Among the salivary proteins and peptides affected by the aging process, S100A8 and S100A9 have been found to participate in the toll-like receptor-4- or RAGE-mediated multiple inflammatory pathways [75]. In fact, S100A8 and S100A9 represent the main protein content of the neutrophils and actively participate in modulating inflammation [151] by also acting as ROS/RNS scavengers. The maintenance of normal salivary function is important for a healthy oral environment. In this context, the study of glycated proteins and peptides in saliva could be, given the low-invasiveness and easy collection of this biofluid, a potential tool to study the aging process as well as disease progression or patient follow-up during treatments [152].

It is relevant to remark that improper sample manipulation can generate an artefactual detection of the non-enzymatic glycation of proteins. For instance, freeze-drying salivary solutions containing glucose and/or fructose can generate artefactual amounts of glycated proteins [22].

### 7.2. Enzymatic Glycosylation

Enzymatic glycosylation is one of the most-common and prominent PTMs, modulating the activity of cellular and extracellular proteins, implicated in complex and articulate physiological processes [153,154] such as cell adhesion, signaling, and cell-to-cell interaction, to cite a few, and many proteins detectable in human saliva are enzymatically glycosylated. The two principal types of protein glycosylation in humans are N- and O-glycosylation, while the C-mannosylation of tryptophan occurs rarely, with its function still not well understood [155].

N-glycosylation occurs as a co-translational modification on the Asn residue at the Asn-X-Ser/Thr sequon, even though some recent glycoproteomic studies evidenced that several N-glycosylation sites do not adhere to this canonical consensus sequence [156]. The glycan structure is not under direct genetic control; rather, it results from the cooperative and competitive interactions between several glycosyltransferases. These enzymes transfer a monosaccharide unit from an activated sugar donor (frequently a nucleotide sugar) to an acceptor (such as an amino acid, a lipid, or another sugar). Consequently, glycosylation must be considered a stochastic rather than a deterministic process. The biological role of glycans is frequently mediated by sugar-binding molecules (lectins), which, upon recognition of specific carbohydrate structures, trigger a broad range of cellular effects, including proliferation, apoptosis, and cell migration [157]. Galectins (beta-galactoside binding proteins) and siglecs (sialic acid-binding immunoglobulin-type lectins) are among these sugar-binding molecules. The activities of specific galectins and siglecs can be inhibited or promoted by non-coding RNAs (ncRNAs), such as those implicated in tumor invasion and cell proliferation [158], as well as by many glycosyltransferases [158]. Indeed, ncRNAs form an extremely complex non-deterministic network of gene expression regulation, and they have a key role in modulating the expression of several glycosyltransferase genes, especially promoting those implicated in the progression and invasion of various kind of tumors [158]. The relevant role of several classes of ncRNAs is nowadays recognized in regulating both physiological and pathological mechanisms. Moreover, the ncRNA aberrant expression appears to be associated with different human disorders, such as reproductive disorders and cancer [159]. The term ncRNAs comprises a wide and heterogeneous group of RNA molecules differing in length, biological function, and cellular localization. ncRNAs are classified, based on their size, into long ncRNAs (lncRNA, >200 nucleotides) and short ncRNAs (<200 nucleotides), which include, among others, microRNAs (miRNAs). Circulating miRNA are largely studied as the next-generation class of diagnostic and prognostic biomarkers. lncRNAs are tissue-specific and can directly interact with other macromolecules, such as DNA, RNA, and protein, and moreover, they can regulate gene expression both at the transcriptional and the post-transcriptional level.

In addition, glycosylation has been demonstrated, on the other hand, to modulate ncRNA expression in some instances. Finally, short ncRNAs have recently been shown to undergo canonical N-glycosylation [160]. These glyco-RNAs bear terminal sialic acid and fucose residues and can interact with sugar-binding molecules, such as siglecs.

Thus, the ncRNA network and glycosylation can be considered as two stochastic mechanisms affecting the role of proteins: the first and the second acting before and after the protein axis biosynthesis, respectively. The N-linked glycans present a common pentasaccharide core to which different types of monosaccharide residues can be linked in different positions, generating diverse classes of branched glycans [161].

O-glycosylation occurs on either Ser or Thr residue exposed on the protein surface. The O-linked glycans can be very simple and are constituted by one or a few monosaccharide residues, such as N-acetyl-galactosamine, mannose, glucose, and fucose. The glycosylation of salivary proteins was studied several years ago using a bottom-up pipeline, identifying about 1500 glycosites [162].

Due to the stochastic mechanism of the synthesis described above, glycoproteins are characterized by high microheterogeneity, because each glycoprotein may exist as a complex family of glycoforms sharing an amino-acid sequence and differing for various features: (a) occupancy of the multiple glycosites; (b) type of glycan (only N-linked, only O-linked, N- and O-linked on the same protein); and (c) composition and stereoisomery of the glycans that can change on the same glycosite.

We were able to characterize using an integrated top-down/bottom-up strategy the structure of six glycoforms of IB-8a (Con 1+), so called because they interact with concanavalin A [163]. MS analyses on the intact glycoproteins before and after N-deglycosylation with the PNGase F and MS/MS sequencing of peptides and glycopeptides from tryptic digests allowed for the structural characterization of the glycan moieties and the polypeptide backbone, as well as the establishment of the glycosylation site at the asparagine residue at the 98th position. Five of the glycoforms carry a biantennary N-linked glycan fucosylated in the innermost N-acetylglucosamine of the core and showed from zero to four additional fucoses in the antennal region. The sixth glycoform carries a monoantennary monofucosylated oligosaccharide. The glycoform cluster was detected in 28 out of the 71 adult saliva specimens analyzed [163].

The level of fucosylation showed interindividual variability, with major relative abundance for the tri-fucosylated glycoform. Non-glycosylated IB-8a (Con 1+) and the variant IB-8a (Con 1−), lacking the glycosylation site for the substitution, have been also detected in human saliva.

The surprising detection of the gPRP protein 3M (encoded by the PRB3 human gene) in the saliva of preterm newborns at an age when the other bPRPs are absent in human saliva [20,25,26] allowed us to purify the 3M protein and to establish the structures of the intact proteoforms before and after N-deglycosylation with peptide-N-glycosidase F and the MS/MS sequencing of peptides obtained after endoproteinase GluC digestion [164]. The heterogeneous mixture of the proteoforms derives from the combination of 8 different neutral and sialylated glycans O-linked to Thr_50_ and 33 different glycans N-linked to various Asn residues at positions 66, 87, 108, 129, 150, 171, 192, and 213 of the 3M protein. In a study carried out to evidence potential salivary diagnostic markers of MuSc [72], we were able to evidence that prolactin-inducible protein exists in two glycosylated proteoforms: one with an N-acetyl-hexosamine residue and the other with a further deoxyhexose moiety. Although the site of modification was not established, these two glycosylated proteoforms were significantly increased in the patients with MuSc with respect to healthy controls. Indeed, altered glycoprotein expression and glycosylation aberrations, consisting of increased glycan size, extra branching of glycan chains, over-sialylation, or over-fucosylation, were associated with many diseases, especially cancer and neurological disorders [165,166]. A recent magistral review [158] evidences the complexity of the relationships between enzymatic glycosylation and other factors and their molecular alterations as potential diagnostic markers in many diseases. Therefore, glycoproteomic studies are of great interest for the development of disease biomarkers. Nonetheless, they are a complex and challenging field of research, requiring the evaluation of the glycoprotein profiling of a biological sample in physiological conditions and the characterization of the qualitative and quantitative changes related to a specific pathological condition [167,168].

## 8. Citrullination

Citrullination is a PTM consisting of the conversion of peptidylarginine to peptidylcitrulline in a calcium-dependent reaction catalyzed by peptidylarginine deiminases (PADs), a family of five isoenzymes (PAD 1–4 and 6), with tissue-specific expression [169,170]. The reaction of the PAD enzyme with the arginine residue of a peptide/polypeptide chain forms an adduct, with the release of ammonia and the subsequent formation of a ketone functional group following the cleavage of the adduct by a water molecule. The result of the deimination reaction is the loss of a positive charge, i.e., the imino moiety, resulting in a generation of a neutral amino acid, citrulline, lacking the strong basic character proper of arginine. It produces the delta mass increase of the protein/peptide molecular mass of +0.9840276 and +0.98476 Da (monoisotopic and average, respectively). Interestingly, the rate of peptidylarginine citrullination by PADs enzymes depends on the amino-acid position along the protein/peptide sequence, with about 80–90% of citrullinated arginines positioned after aspartic acid residues. Arginines close to glutamic acid or to the N-terminal sequence trait are also largely citrullinated [169,170,171].

The citrullination affects the physico-chemical properties of a protein because, at a neutral pH, the positive charge of arginine is lost by the modification and causes changes in the overall charge and charge distribution of the protein, altering the isoelectric point, ionic bonds, protein structure, activity, and protein–protein interactions [169,170,171]. The increase in hydrophobicity produced by citrullination allows to easily distinguish the un-modified from the modified form of a small protein or a peptide by reverse-phase liquid chromatographic separations. In addition, because citrulline is not included in the list of natural amino acids incorporated into proteins, it was hypothesized to possibly induce an immune response, and it was suggested to deeply investigate its role in autoimmune inflammatory diseases such as rheumatoid arthritis (RA) [172]. In fact, citrullination was studied in relation to various physio-pathological processes in addition to RA, such as apoptosis, multiple sclerosis, Alzheimer’s disease, psoriasis, systemic lupus erythematosus, periodontitis, COVID-19, cancer, and thromboembolism [169,170,171]. Citrullination has been the topic of a very recent review that outlines the diagnostic as well as therapeutic approaches based on this PTM and the future perspectives of “citrullinome” characterization and disclosure [172]. Studies and clinical trials are nowadays based on the detection of anticitrullinated protein antibodies as a high-specificity tool for RA diagnosis, before clinical evidence [173,174].

Several are the proteins that are substrates of PAD enzymes and that can exhibit citrullination PTM in physiological, as well as in inflammatory, pathological states, and they include filaggrin, keratin, fibronectin, actin, tubulin, vimentin, glial fibrillary acidic protein, and histones [173]. PAD 2 is the isoenzyme acting in salivary and secretory glands, as well as in other cell types and tissues, targeting myelin basic protein, C-X-C motif chemokine 10 and 11, vimentin, actin, glial fibrillary acidic protein, S100-A3, and histones H3 and H4 [173]. The importance of the detection of anti-citrullinated protein antibodies (ACPAs) in serum for the diagnosis of RA [173,175] has increased the interest of their application to saliva analysis for the characterization of citrullinated proteins.

Yasuda et al. identified citrullinated cytokeratin 13 in saliva samples of RA patients and healthy subjects by two-dimensional electrophoresis, silver staining, and immunoprecipitation Western blotting [175]. In the same paper, the origin of the pre- or post-secretion of citrullinated cytokeratin 13 in saliva is also discussed based on calcium concentration data inside salivary gland cells and secreted saliva, since PAD enzyme catalysis is calcium-dependent. According to the Genotype-Tissue Expression database, PAD 1–4 and six calcium-binding enzymes are reported to be expressed in human minor salivary glands [176]. Citrullination could take place either in saliva, i.e., post-secretion because the biofluid contains calcium levels proper to PAD enzyme activity, or intracellularly, dependent on the calcium concentration increasing. Indeed, intracellular citrullination of histones and other proteins was recognized [177]. Citrullination of salivary proteins could therefore occur either intracellularly, followed by the secretion of the modified protein, or extracellularly in the biofluid, after secretion.

A recent paper compared the levels of citrullinated proteins levels in the saliva of RA patients with respect to healthy controls, also studying their correlation with periodontal status and a temporomandibular joint-associated disorder [178]. Confirming previous data [175], no differences in citrullinated protein levels were found between RA patients and healthy controls, apart from the differences in periodontal status observed between groups. However, in RA, the salivary anti-cyclic citrullinated peptide levels were in correlation with periodontal disease staging, considering that the bacteria involved in its pathogenesis produce non-physiological citrullination by a family of PAD enzymes very similar to human PADs [179].

One study investigated the influence of inflammation from periodontal disease using RA-model mice analysis in serum and saliva samples of both anti-citrullinated proteins (using antibody analysis) and citrullinated proteins (using gel electrophoresis) [180]. Saliva and serum showed similar results, evidencing protein citrullination around the 55 kDa molecular-weight electrophoretic band. Periodontitis exacerbated RA symptoms, indicating a relationship between the *Porphyromonas gingivalis* periodontitis bacterial infection and RA.

Patients affected by Sjögren’s syndrome have been found to be positive for ACPAs and showed increased levels of the PAD 2 enzyme, suggesting a possible role of citrullination in the disease that could be responsive to autoantigens triggering [180]. However, in the same study, it was outlined that only approximately 7.5% of Sjögren’s syndrome patients are positive for ACPAs. Despite the poor knowledge of the protein pattern that led to citrullination in saliva, the indications of the high potential of this biofluid are clear in terms of the development of new high-specificity and -sensitivity diagnostic tools of low invasiveness targeting selected citrullinated proteins in different pathologies. However, deeper research still needs to be conducted to decipher the saliva citrullinome associated with physiological as well as pathological states and to clearly understand where the modification occurs, i.e., inside salivary gland cells, extracellularly, or both. To the best of our knowledge, combined top-down/bottom-up proteomic platforms have never been applied to the study of the saliva citrullinome. Their application coupled with high-resolution mass-spectrometry detection could finally characterize the proteins subjected to citrullination in saliva in a wide molecular range and abundance, and, especially, could localize the modification inside the sequence and better define its molecular features.

## 9. N-Terminal Modifications

The N-terminal of a polypeptide chain is a structural site that must often be modified and protected during ribosome synthesis. The positive charge of the terminal amino group can be an obstacle to the proper structural folding of the protein, which starts in the nascent protein [181]. The most common modification in humans is the excision of the initiatory methionine (iMet), which is commonly carried out by enzymes called methionine aminopeptidases (MAPs). In any case, the polypeptide chain, with or without iMet, can be submitted to N-terminal acetylation induced by enzymes called N-terminal acetyltransferases (NATs). To date, 12 NATs have been identified, possessing different compositions, substrate specificities, and modes of regulation. In humans, NATs are present as a group of six enzymes (called from NAT-A to NAT-F), with slightly different specificities [182].

Another possibility, less common, of N-terminal modification occurs when the terminal residue is either a glutamic acid or a glutamine, which can generate a cyclic amide, called pyroglutamic N-terminus, with the loss of a molecule of water (Glu) or ammonia (Gln), respectively. This PTM can proceed spontaneously, at reasonable rates, or by the action of enzymes called glutaminyl cyclases, in a reaction commonly much faster with N-terminal glutamines than with glutamate residues [183].

Our top-down mass-spectrometry pipelines allowed us to verify that these three PTMs are largely detectable in human saliva, while C-terminal modifications are rarely detectable, probably because at the end of their synthesis the proteins have assumed a folding close to the functional conformation. A few minor examples of C-terminal modifications of human salivary proteins will be reported at the end of this section.

### 9.1. Excision of Initiatory Methionine and Nt-Acetylation

It is relevant to outline that when the methionine residue is at the first position of a leader peptide, in turn responsible for the docking of the protein to the ribosome at the endoplasmic reticulum, acetylation is not carried out. This is observed for cystatins A, B, and S-type (S, SA, and SN), and all the PRPs, suggesting the absence of NATs in the cisternae of the cytoplasmic reticulum. In all other proteins, the action of MAPs is not mandatory and seems to depend on the type of the residue present in the second position in the sequence. Therefore, Nt-acetylation may occur either on the iMet or on the first residue after iMet excision by MAPs. The N-termini of proteins with small amino-acid residues (Ser, Ala, Thr, Val, Gly, and Cys) in the second position are mostly processed by MAPs, and the newly generated N-termini may be acetylated by NAT-A, as observed for the serine residue of thymosins β4 and β10; of S100A7; and of the two proteoforms of small proline-rich protein 3 [87,184,185,186]. The N-termini of proteins with larger amino-acid residues in the second position are not cleaved by MAPs but potentially acetylated directly on the iMet by a variety of NATs, depending on the N-terminal sequence. NAT-B potentially acetylates Met-N-termini when the second residue is Asp, Glu, or Asn. NAT-C potentially acetylates Met-N-termini when the second residue is a hydrophobic amino acid (mainly Leu, Ile, and Phe). Hydrophobic terminals are also recognized by NAT-E and NAT-F, suggesting that redundancy in activity exists between NATs [187]. Moreover, NAT-F showed a broader specificity than NAT-C in acetylating Met-N-terminals when the second residue was Met, Lys, or Gln, and the potential to acetylate the same N-terminals was recognized by NAT-A in proteins in which iMet has not been cleaved [188]. Examples of human salivary proteins are represented by cystatin A, which has an isoleucine residue in position 2 and is probably acetylated on Met1 by NAT-C (or NAT-F), and cystatin B, which has a second methionine residue in position 2 and is acetylated on Met_1_ probably by NAT-F [57,87].

A peculiar example concerns the acetylation of S100A9, which among the five N- terminal residues has two Met residues—one at position 1 and the other at position 5 (MTCKM…). Because of this N-terminal sequence, MAP catalyzes the cleavage not only at the level of the first Met residue, generating the long proteoform (1–113 residues), but also at the level of the fifth Met, giving rise to the short proteoform (1–109 residues), with the percentages of the two proteins being similar in human saliva [25,87]. The Cys_3_ residue, present only in the long form, is responsible for the formation of the S-cysteinyl and S-glutathionyl derivatives of S100A9, described in Section 5. Since these four proteoforms of S100A9 can also be phosphorylated in the penultimate threonine residue of the sequence, the number of possible proteoforms of S100A9 detectable in human saliva is eight [188]. As far as we are aware, no information exists about the specific function of these different proteoforms of S100A9.

### 9.2. Pyroglutamic Acid Modification

As above reported, several human salivary proteins undergo the N-terminal cyclization of glutamine and glutamic acid as a protection for the N-terminal amino group. This PTM has been characterized for all the proteoforms of aPRPs (PRP-1 and PRP-3 types) [90,189], bPRPs (II-2, IB-1), all the gPRPs [23], and the P-B peptide [41]. It is relevant to remark that although this PTM allows for the integrity of the protein structure by protecting it from the action of aminopeptidases [190], on the other hand, it significantly alters its hydrophobicity and solubility [183], facilitating protein aggregation. For this reason, many authors hypothesized that it plays a relevant role in several amyloidopathies [191]. Indeed, experiments carried out in a mouse model using glutaminyl cyclase inhibitors demonstrated a reduced level of pGlu-modified Aβ peptides that appeared to attenuate Alzheimer’s disease [192]. Nonetheless, the enzyme does not seem essential for the cyclization, which according to Bersin and colleagues [193], is spontaneous and is also observed in the absence of the enzyme.

## 10. C-Terminal Modifications

Except for the removal of C-terminal residues by carboxy-peptidases, described in Section 2.1.2, only three C-terminal modifications were detected during our top-down experiments. One is the formation of a cyclic anhydride bond for the loss of one water molecule from two carboxylic groups in the presence of a C-terminal Glu residue of the fragment 1–26 of β-globin; the second is the formation of one cyclic ester for the loss of a water molecule from the C-terminal serine of the fragment 1–42 of P-C peptide; and the third is the formation of a cyclic N-substituted amide for the loss of one water molecule from the C-terminal glutamine of the fragment 1–41 of the P-C peptide.

## 11. Conclusions

This review, which provides many answers regarding the complexity of the PTMs that human salivary proteins undergo, nevertheless prompts even more questions regarding the roles of many of them.

The first question concerns the possible functional role played by the products generated by the multiple proteolytic cleavages to which the components of almost all protein families are subjected.

It is relevant to underline that they could be evidenced only by a top-down strategy. Since evolution is addressed towards the selection of molecular events relevant to the functions of cells, organs, and tissues, particularly puzzling is the functional meaning of the cleavages that undergo acidic and basic PRPs, as well as the challenging role of the P-B peptide. Particularly challenging is defining the role of the myriad recurrent fragments generated from bigger salivary proteins by exogenous proteinases. Therefore, the development of new strategies to study the biological role of the fragments is demanding.

The role of almost all the other PTMs described is also obscure. Proteomic searches, devoted to the discovery of new disease biomarkers, are observational studies, supported by powerful statistical tools; they are able to suggest potential biomarkers but not able to suggest their specific physio-pathological roles. Therefore, new experimental designs are needed to help researchers connect the information obtained on structural modifications and the role of these changes in health and disease. This knowledge is fundamental for the biotechnological utilization of biologically active salivary proteins [194].

The recent pandemic evidenced the utility of saliva as a human biofluid for painless, non-invasive, free, at-home diagnostic purposes [1,195,196]. Indeed, saliva is currently also used for the fast check of illicit drug abuse on the road [196]. We hope that this review could be a stimulus for further investigations and clinical applications.

## Figures and Tables

**Table 1:** Some terminal losses detected in salivary peptides/proteins.

Protein and Peptides	C-Term. Cleavage
aPRP Pa-dimer	…QSP↓Q
PRP3/PRP4/PIF-f	…RPP↓R
P-C (or IB-8b)	…QS↓P↓Q
bPRP II-2	…RSP↓R
bPRP IB-1	…RSP↓R
bPRP PF (or IB-8c) precursor	…RSA↓R
bPRP PE (or IB-9)	…RSP↓R

**Table 2:** Phosphorylation sites recognized by Fam20C of human-specific secretory proteins.

Protein/Peptide	Partial Sequence	Consensus	Refs.
Statherin (Ser_2_, Ser_3_)	DSSEEKF…	SXE	[41]
Histatin 1 (Ser_2_)	DSHEKR…	SXE	[49]
Cystatin S (Ser_3_)	SSSKEENR…	SXE	[57,58,59,83]
Cystatin S1 (Ser_1_)	SSS_(phos)_KEENR…	SXS_(Phos)_	“
aPRP (Types 1 and 3) (Ser_8_)	…DEDVSQEDV….	SXE	[9]
aPRP (Types 1 and 3) (Ser_22_)	…GGDSEQFIDEER…	SX(_3/4_)(E/D/S(_phos_))_3_	“
aPRP (Types 1 and 3) (Ser_17_)	…LVISDGG DS(_phos_)EQFI…	SX(_3/4_)(E/D/S(_phos_))_3_	“
bPRP II-2 (Ser_8_)	…NEDVSQEESPS…	SXE	[23]
bPRP IB-1 (Ser_8_)	…NEDVSQEESPS…	SXE	“
gPRP Gl-2 or PRP-3M (Ser_8_)	…NEDVSQEESPS…	SXE	“
gPRP Gl-1 or PRP-3L (Ser_8_) ^1^	…NEDVSQEESPS…	SXE	“
gPRP Gl-3 or PRP-3S (Ser_8_) ^1^	…NEDVSQEESPS…	SXE	“
gPRP Glycosyl. Protein A (Ser_8_) ^1^	…SEDVSQEESLFL…	SXE	“
gPRP II-1 (Ser_8_) ^1^	…SEDVSQEESLFL…	SXE	“
gPRP Cd-IIg (Ser_8_) ^1^	…SEDVSQEESLFL…	SXE	“

^1^ Hypothetical (by similarity).

## Data Availability

Not applicable.
